# Validity and reliability of the Cold Discomfort Scale: a subjective judgement scale for the assessment of patient thermal state in a cold environment

**DOI:** 10.1007/s10877-013-9533-7

**Published:** 2013-12-06

**Authors:** Peter Lundgren, Otto Henriksson, Kalev Kuklane, Ingvar Holmér, Peter Naredi, Ulf Björnstig

**Affiliations:** 1 Division of Surgery, Department of Surgery and Perioperative Sciences, Umeå University, Umeå, Sweden; 2 The Thermal Environment Laboratory, Division of Ergonomics and Aerosol Technology, Department of Design Sciences, Faculty of Engineering, Lund University, Lund, Sweden

**Keywords:** Hypothermia, Prehospital trauma care, Emergency medical services, Reliability, Validity, Subjective judgement scale, Thermal comfort

## Abstract

Complementary measures for the assessment of patient thermoregulatory state, such as subjective judgement scales, might be of considerable importance in field rescue scenarios where objective measures such as body core temperature, skin temperature, and oxygen consumption are difficult to obtain. The objective of this study was to evaluate, in healthy subjects, the reliability of the Cold Discomfort Scale (CDS), a subjective judgement scale for the assessment of patient thermal state in cold environments, defined as test–retest stability, and criterion validity, defined as the ability to detect a difference in cumulative cold stress over time. Twenty-two healthy subjects performed two consecutive trials (test–retest). Dressed in light clothing, the subjects remained in a climatic chamber set to −20 °C for 60 min. CDS ratings were obtained every 5 min. Reliability was analysed by test–retest stability using weighted kappa coefficient that was 0.84 including all the 5-min interval measurements. When analysed separately at each 5-min interval the weighted kappa coefficients were was 0.48–0.86. Criterion validity was analysed by comparing median CDS ratings of a moving time interval. The comparison revealed that CDS ratings were significantly increased for every interval of 10, 15, and 30 min (*p* < 0.001) but not for every interval of 5 min. In conclusion, in a prehospital scenario, subjective judgement scales might be a valuable measure for the assessment of patient thermal state. The results of this study indicated that, in concious patients, the CDS may be both reliable and valid for such purpose.

## Introduction

Admission hypothermia is an independent risk factor associated with worse outcome and higher mortality in trauma patients [1–6]. Initial actions to reduce cold exposure and prevent further heat loss are therefore important and integrated aspects of prehospital primary care [7–11]. Consequently, it is important to have accurate measures for the evaluation of patient thermoregulatory state, both upon arrival of the rescue team and during patient treatment and evacuation. In the field, especially in harsh ambient conditions this is often hard to achieve. Although of utmost clinical importance, measuring body core temperature as well as skin temperature might be difficult [9] and measuring oxygen consumption for the assessment of shivering is, in most clinical scenarios, not possible. Simplified hypothermia staging protocols that consider level of consciousness and the presence or absence of shivering have been developed to deal with such practical complexities [12]. Cold induced stress response also renders thermal discomfort, which might increase the experience of pain and anxiety, even in normothermic patients [13–17]. To address such aspects of primary care, complementary measures, such as subjective judgement scales for the assessment of patient thermal state might be of considerable importance both in the initial assessment and for evaluation of the treatment provided. It is of utmost importance that those subjective judgement scales are reliable and valid.

The most common single item judgement scales are Visual Analouge Scales (VAS), Numerical Rating Scales (NRS) and Verbal Rating Scales (VRS). In clinical practice such scales are frequently used and have been shown valid and reliable for the assessment of pain [18, 19]. The international standard BS EN ISO 10551:2001 outlays general principles for construction of subjective judgement scales for the assessment of the influence of the thermal environment [20]. There are, however, to the authors’ knowledge, no previous studies on reliability and validity of such psychometric methods for the assessment of the influence of the thermal environment in more extreme ambient conditions.

In accordance with the basic principles stated in the international standard [20] and with some modifications to increase usefulness in a prehospital rescue scenario we have designed an NRS, the Cold Discomfort Scale (CDS), for the assessment of patient thermal state in a cold environment [17]. The objective of this study was to evaluate this NRS in healthy subjects exposed to −20 °C for 60 min. The NRS was evaluated for reliability, defined as test–retest stability; and criterion validity, defined as the ability to to detect a difference in cumulative cold stress over time.

## Methods

### Design, settings, and subjects

The study was conducted in October and November 2011 at the Thermal Environment Laboratory, Lund University, Sweden. Thirteen male and nine female volunteers participated. The age, weight, and height of the subjects were 23.3 ± 4.4 years, 72.7 ± 15.3 kg, and 178.9 ± 9.6 cm respectively (mean ± SD). Subjects were cardiopulmonary healthy and were not taking regular medication and did not have history of local cold injuries. No subjects were habitual smokers or abusers of narcotics. Written informed consent was given by all subjects. Ethical approval was given by the Regional Ethical Review Board in Umea.

The study protocol was designed as a test–retest where subjects were exposed to −20 °C for 60 min evaluating the reliability and criterion validity of the CDS. Reliability refers to a measure’s lack of errors of measurement. Validity can be divided into content, construct, and criterion validity, where criterion validity refers to a measure’s association with one or more outcome criteria. In this study criterion validity was defined as the ability to detect a change in cumulative cold stress over time based on the prevailing ambient conditions. Reliability was defined as test–retest stability. All subjects conducted two identical trials on two separate occasions, approximately 1 week apart at about the same time of day. During the twenty-four hour period prior to the trials subjects did not smoke or drink alcohol and had a night rest of minimum of 6 h. Additionally the subjects were instructed to avoid physical excertion. Diet was not modified but they all had regular meals.

### Monitoring

Cold discomfort was monitored every 5 min using the CDS, where the subjects assess the thermal state of their whole body, not specific body parts. Subjects provide integer values from 0 to 10, where 0 indicates no experience of cold and 10 indicates unbearable cold. Subjects were asked the following question:

On a scale from 0 to 10, where 0 means not feeling cold in any way and 10 means feeling unbearably cold: How cold do you feel right now?

To ensure that there was no risk of local cold injuries, finger and toe temperatures were continuously monitored using thermistors (Rhopoint Components Ltd, UK, accuracy ± 0.2 °C) taped to the left ring finger and the left index toe.

Ambient air temperature was continuously monitored using three sensors (PT 100, Pico Technology Ltd, UK, accuracy ± 0.03 °C) positioned in level with the supine subject, adjacent to the ankles, mid-trunk, and the head.

### Protocol

Subjects were dressed in lightweight two-piece thermal underwear, a fleece cap, two pairs of gloves, two pairs of woollen socks, and an outer foot cover. Insulation of hands and feet were reinforced to avoid the risk of local cold injuries. At first, subjects sat quitely at an ambient temperature of about 21 °C for 15 min for baseline data collection. They then entered the climatic chamber (2.4 × 2.4 × 2.4 m), set to −20 °C, and lay down in a supine position on a foam mattress. One of the physicians responsible for the study (P.L or O.H) accompanied the subject in the cold chamber during the whole trial and every 5 min the subjects were asked to express their thermal state according to the CDS. After 60 min of cold exposure the trial was completed and subjects exited the cold chamber.

### Data analysis

As the CDS comprises ordinal data non-parametric statistics were used. Reliability of the CDS was analysed for test–retest stability, using weighted (quadratic difference) kappa coefficient [21], comparing median CDS ratings between the two trials, including all the measurements made every 5 min and also separately for every 5-min interval. StatXact 9 software (Cytel inc., Cambridge, MA, USA) was used for this analysis.

Criterion validity was analysed by comparing median CDS ratings over moving intervals of 5, 10, 15, and 30 min (5–10, 10–15 min, etc.; 5–15, 10–20 min, etc.; 5–20, 10–25 min, etc.; 5–35, 10–40 min, etc.) using Wilcoxon Signed Ranks test. Statistical significance was defined as *p* < 0.05 and after correction for 36 multiple comparisons according to Bonferroni as *p* < 0.001. Pre-study calculations indicated a minimal sample size of 18 to detect a median difference in CDS ratings of 2 or more (interquartile range, IQR; 2) with 80 % statistical power at an α-level of 0.05. SPSS 18.0 software (SPSS inc., Chicago, IL, USA) was used for this analysis.

## Results

All of the scheduled 44 trials were conducted according to the study protocol. The ambient air temperature for the first set of trials (test) was −19.3 ± 0.2 °C (mean ± SD) and for the second set of trials (retest) −19.1 ± 0.6 °C with no statistical significant difference between the trials. Wind speed for all trials was 0.2 ± 0.0 m/s (mean ± SD). Skin temperature of the left ring finger and left index toe never went below 8 °C for any of the subjects.

**Fig. 1:**
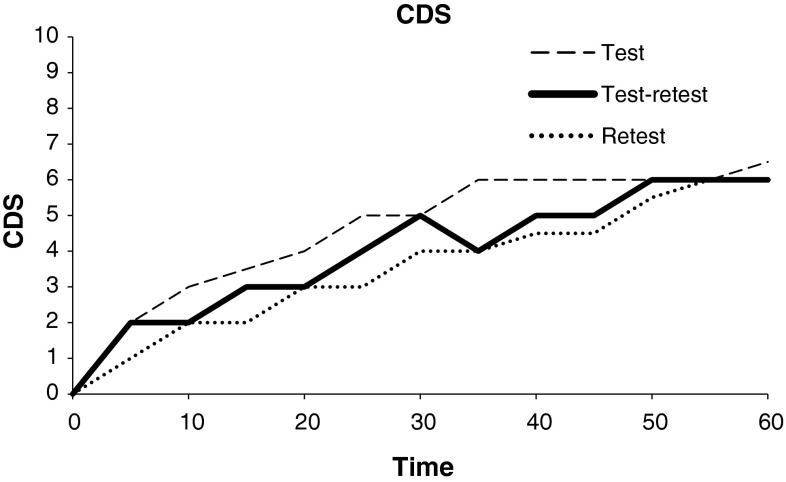
CDS ratings measured every 5 min in 22 healthy subjects. Two consecutive trials of cold exposure in −19.2 °C still wind conditions. Median CDS ratings of test (n = 22), retest (n = 22) and merged median CDS ratings of test and retest (n = 22) are presented

Median CDS ratings increased from 0 (interquartile range, IQR; 0–0) during baseline to 7 (IQR; 5–7) at the end of the first set of trials (test) and from 0 (IQR; 0–0) to 6 (IQR; 5–7) during the second set of trials (retest) (Fig. 1).

Reliability analysis by test–retest stability revealed that weighted kappa coefficient was 0.84 including all the measurements made every 5 min and ranged from 0.48 to 0.86 when analysed separately at each 5-min interval (Table 1).

**Table 1:** Test, re-test and merged (test and re-test) median CDS ratings by volunteer subjects (n = 22) at 5 min intervals during 60 min of cold exposure in −19.2 °C wind still conditions

Time (min)	Test^a^ (n = 22)	Re-test^a^ (n = 22)	Merged^a^ (n = 22)	Weighted kappa coefficient^b^ (n = 22)
5	2 (1.25–3)	1 (1–2)	2 (1–2.25)	0.56 (0.25–0.86)
10	3 (2–3)	2 (1–2)	2 (2–3)	0.48 (0.20–0.77)
15	3.50 (3–4)	2 (1.25–3.75)	3 (2–4)	0.56 (0.31–0.81)
20	4 (3.25–4)	3 (2–4)	4 (2–4)	0.60 (0.38–0.83)
25	5 (4–5)	3 (2.25–4.75)	4 (3–5)	0.53 (0.30–0.76)
30	5 (4–6)	4 (3–5)	5 (3––6)	0.68 (0.48–0.87)
35	6 (4–6)	4 (3–5)	5 (3.75–6)	0.64 (0.40–0.88)
40	6 (4–6)	4.5 (6–4)	5.5 (4–6)	0.70 (0.49–0.90)
45	6 (4.25–6)	4.5 (6–4)	6 (4–7)	0.72 (0.51–0.92)
50	6 (5–7)	5.5 (5–7)	6 (5–7)	0.76 (0.57–0.96)
55	6 (5.25–7)	6 (5–7)	6 (5–7)	0.86 (0.72–1.0)
60	6.5 (5.25–7)	6 (5–7)	6 (5–7)	0.85 (0.81–0.99)

Values are median (IQR)^a^ and weighted kappa coefficient (95 % CI)^b^

Reliability (test–re-test stability) presented moderate to very good agreement (weighted kappa coefficient 0.48–0.86)

Criterion validity (comparing merged CDS ratings over moving time intervals) presented a significant increase (Wilcoxon Signed Ranks test) in CDS ratings for each 10, 15 and 30 min interval (*p* < 0.001) but not for every 5 min interval

Criterion validity analysis by comparing median CDS ratings (n = 22) over moving time intervals of 5, 10, 15, and 30 min revealed that CDS ratings were significantly increased for every time interval of 10, 15 and 30 min (*p* < 0.001), but not for every time interval of 5 min (Table 1).

## Discussion

### Overview

In a laboratory setting the test–retest stability of median CDS ratings over the 60 min of cold exposure was 0.84 (very good agreement) when all the measurements made every 5 min were included in the analysis and 0.48–0.86 (moderate to very good agreement) when analysed separately at each 5-min interval [21]. The CDS was significantly sensitive to detect a difference in cumulative cold stress for time intervals of 10, 15, and 30 min throughout the whole 60 min of cold exposure.

### Reliability

It is always difficult to achieve identical conditions in a test–retest design when measuring subjective parameters. Even if all conditions are the same, the subject might react differently to the same level of cold exposure on two different occasions. There might also be an element of adaptation that could either increase or decrease the sensitivity to exposure. CDS ratings were generally somewhat higher in the first trial compared to the second trial, and this difference might be a result of a decreased sensitivity to the cold exposure from previous experience, therefore the subject might be less anxious about cold exposure the second time compared to the first time. However, test–retest stability was still very good when all the measurements every 5 min were included and moderate to very good when analysed separately at each 5-min interval.

### Validity

The results revealed that CDS ratings were statistically significant increased for every interval of 10, 15, and 30 min, wich means the CDS was valid for detecting such a difference in cumulative cold stress. However, CDS ratings were not statistically significant increased for every 5-min interval, which means the CDS was not valid for detecting such small differences in cumulative cold stress. Also, during the last 20 min it appears that CDS ratings were not increasing at the same rate as during the first 40 min which might be an indication of a limitation to detect differences in cumulative cold stress because of subject habituation to ambient conditions when cold exposure is protracted.

### Practical implications

In addition to objective measurements, including simplified hypothermia staging protocols [12], subjective judgement scales might be an important adjunct for the assessment of patient thermal state in a cold environment. When using subjective judgement scales, early recognition of cold stressed patients might be improved and such scales may, therefore, aid in evaluating the risk of developing hypothermia. Another important aspect of primary prehospital care is thermal comfort. Many resources and much effort are invested in optimising medical care, including pain relief, but thermal comfort is easily and often forgotten. Reliable and valid subjective judgement scales for the assessment of patient thermal state in a cold environment is therefore necessary for improving prehospital medical care. This study indicates that the CDS is reliable and valid for such purpose.

The general principles for constructing subjective judgement scales for the assessment of the thermal environment recommend symmetrical 7–9-degree rating scales comprising a central indifference point and two times 3 or 4 degrees of increasing intensity for both hot and cold. Subjective judgement scales used in prehospital as well as hospital medical care most commonly ranges from 0 to 10, for example when assessing pain intensity using the VAS, and therefore we considered a similar range of the CDS would be more easily understood by patients and, also very important, more familiar to the rescue personel. Subjective judgement scales with differing ranges could be confusing for patients and medical personel alike. Furthermore, because we are only interested in cold exposure, we think it better to simplify the scale to be assymetrical, describing only cold. In the litterature [20, 22] there is a distinction between perception/thermal sensation and affective assessment/(dis)comfort. The CDS does not differentiate between thermal sensation and (dis)comfort. This design enables rescue personel to give short, concise instructions to patients when obtaining data instead of explaining the different definitions of perception versus affective assessment. We think these modifications to international standard instructions give the CDS advantages in practical use in a prehospital rescue scenario.

### Limitations and further research

Subjective judgement scales used as a tool for the assessment of patient thermal state, are, of course, limited to conscious patients, not suffering from any major distracting injury. To the authors’ knowledge this is the first study evaluating reliability and criterion validity of a subjective judgement scale for the assessment of patient thermal state in an extreme cold environment. Considering the small study population, the limited time period for cold exposure, and limited ambient conditions; further studies to confirm these results are encouraged. Furthermore, it would be desirable to validate the scale in a large clinical trial where varying ambient conditions and various clinically important confounding factors are considered. Measuring objective parameters, such as respiratory rate, heart rate, body core, and skin temperature; thereby providing the ability to analyse construct validity is also necessary to fully validate the scale.

## Conclusion

In a prehospital rescue scenario subjective judgement scales might be a valuable measure for the assessment of patient thermal state. The results of this study indicated that, in concious patients, the CDS may be both reliable and valid for such purpose.
